# Plasma-Rich Fibrin—Regenerative Material in Tympanic Membrane Surgery

**DOI:** 10.3390/medicina59071292

**Published:** 2023-07-13

**Authors:** Cristina Tiple, Magdalena Chirila, Stefan Cristian Vesa, Mirela Cristina Stamate

**Affiliations:** 1 ENT Department, County Emergency Clinical Hospital, 400006 Cluj-Napoca, Romania; cristinatiple@yahoo.com (C.T.); mirelacristinastamate@gmail.com (M.C.S.); 2 Department of Pharmacology, Toxicology and Clinical Pharmacology, Iuliu Hatieganu University of Medicine and Pharmacy, 400012 Cluj-Napoca, Romania; stefanvesa@gmail.com

**Keywords:** ear drum perforation, platelet rich fibrin, tympanoplasty

## Abstract

*Background and Objectives*: Platelet-rich fibrin (PRF) membrane plays an important role in cell proliferation and aids in healing. This study aimed to assess the safety and efficacy of the addition of PRF to the graft in tympanoplasty. *Materials and Methods*: A retrospective study was conducted involving 47 patients with chronic dry eardrum perforation, who were candidates for different types of tympanoplasty (type I-IV). The study took place in the ENT department, County Emergency Clinical Hospital of Cluj-Napoca. In group 1 (27 patients) tympanoplasty was performed with a cartilage graft, while in group 2 (20 patients) a cartilage graft was used with the addition of a PRF membrane. The PRF clot was extracted and transformed into a thin membrane. Postoperative evaluation included otoendoscopy and otomicroscopy at 1, 3, 6, and 12 months after surgery, as well as pure-tone audiometry at 12 months. *Results*: Postoperative follow-up at 1, 3, 6, and 12 months showed a higher rate of graft survival in the PRF group than in the non-PRF group. At the 12-month mark, a successful outcome was observed in 95.0% of patients in the PRF group, while the success rate in group 1 was 70.4% (*p* < 0.05). The postoperative hearing threshold value was statistically significantly lower in the group with PRF, compared to the non-PRF group, being 18.4 ± 10.4 dB and 27.6 ± 16.2 dB (*p* < 0.001), respectively. Although the postoperative air-bone gap value did not differ significantly between groups, there was a greater improvement in the PRF group (*p* < 0.7). The PRF was well tolerated, and the incisions healed perfectly. *Conclusions*: The PRF membrane increases the rate of autograft survival and is therefore an effective material for patients with chronic perforations of the tympanic membrane.

## 1. Introduction

The purposes of closing chronic dry perforations of the tympanic membrane are to improve hearing and prevent middle ear infections [1]. In current middle ear surgery, perichondrium, cartilage, and fascia are commonly used materials for repairing a chronically perforated eardrum. These tissues can be easily harvested from the surgical area and their biological characteristics are useful, especially in the early stage of the healing process [2]. Using autologous grafts, obtained from the patient’s own tissues, offers several advantages in terms of biocompatibility and safety compared to materials of animal origin [3]. Based on the existing literature on autologous platelet rich fibrin membrane (PRF), we have opted to incorporate PRF as a supportive material for reparative middle ear surgery.

Though PRF was initially developed in 1970 [4], it was Choukroun who first described its preparation in 2001 [5]. Dohan reported that PRF forms a slowly polymerized fibrin network containing cytokines, glycanic chains, and structural glycoproteins [6]. Since then, PRF has been widely used in various medical fields for soft tissue regeneration, augmentation, and wound healing. Its applications extend to dental and maxillofacial surgery, where it has been utilized to accelerate endosseous wound healing [7]. Furthermore, PRF has found applications in ophthalmology for conditions such as dry eye, graft-versus-host disease, persistent epithelial defects, corneal ulcers or perforations, burns, and post-LASIK syndrome [7]. Additionally, PRF has been reported as a therapeutic solution in gynaecology for wound healing after caesarean delivery, vulvovaginal atrophy, and benign cervical ectopy. Its applications have also been explored in reproductive medicine and the treatment of erectile dysfunction [8,9,10].

Studies in ENT reported the use of PRF, particularly in ear surgery for repairing eardrum perforation [7,11]. PRF serves as a resorbable membrane that provides mechanical and inflammatory protection to the tympanic membrane [12], thereby improving the post-operative outcomes of ear surgery [13]. One of the advantages of PRF is its easy accessibility, allowing for unlimited utilization in myringoplasties, unlike the limited availability of cartilage, perichondrium, or temporalis fascia [14]. Additionally, as PRF is an autologous membrane, the risk of immunogenic reaction, disease transmission and graft failure are minimal [14,15].

The aims of this study were to evaluate the safety and efficacy of incorporating PRF into the graft during tympanoplasty. To assess its efficacy, we focused on evaluating the anatomical (graft survival) and functional (hearing improvement), associated with the use of autologous PRF in graft uptake.

## 2. Materials and Methods

### 2.1. Study Design

The study was conducted in the ENT department of our institution from August 2019 to September 2020. It was designed as a retrospective study and included adult patients with chronic dry eardrum perforation who were candidates for various types of tympanoplasty (tympanoplasty type I-IV). The patients underwent either a cartilage graft or a cartilage graft combined with the PRF graft.

Inclusion criteria: adult patients diagnosed with chronic eardrum, which had been dry for at least 6 months, without any clinical and paraclinical signs of active infection, without history of otological surgery or history of radiation to the head and neck region were included in the study. Patients with diabetes or hypertension were included, provided that their conditions were well controlled with therapy.

Exclusion criteria: unwillingness to participate or refusal of the PRF grafting, presence of congenital malformations in the head area, presence of wet perforation or active infection, and comorbidities that contraindicate ear surgery. Additionally, patients on chronic antiplatelet or anticoagulant treatment, as well as those in whom the procedure for preparing PRF failed, were also excluded from the study.

All 20 patients who underwent cartilage graft with the addition of a PRF membrane were included in the study (PRF group). Additionally, there were 96 patients who underwent tympanoplasty with a cartilage graft. We used stratified randomization, considering the age of PRF group patients, and included 27 patients who underwent tympanoplasty with a cartilage graft in the control group. Each patient underwent anamnesis and comprehensive clinical examination, which included an assessment of the entire ENT region. Additionally, otological examination was performed. Otomicroscopy was utilized to evaluate the characteristics of the eardrum perforation, such as size, location, and the presence of otorrhea or cholesteatoma. Audiological assessment, specifically pure-tone audiometry, was conducted to measure the air-bone gap and hearing threshold. Otomicroscopy was employed to categorize the perforations based on their size, classifying them as small (less than 50% of the total surface area), medium (50–75% of the total surface area), or large (more than 75% of the total surface area).

Postoperative evaluation included otoendoscopy and otomicroscopy at 1, 3, 6, and 12 months after surgery. These examinations aimed to assess the healing process of the eardrum, verify the integrity of the graft, and identify any presence of discharge, residual perforation, granulation tissues, cholesteatoma recurrence, or residual. Additionally, pure-tone audiometry was conducted at the 12-month mark to quantify the postoperative air-bone gap and measure the gain in hearing threshold.

All the evaluations (otoendoscopy, otomicroscopy, hearing assessment) were performed by the same team, under the same conditions, using the same equipment on both ears. The hearing investigation was performed in soundproof rooms, using an Interacoustic Audiometer Affinity Suite. Tonal sounds were used with standard frequencies (0.125, 0.25, 0.5, 1, 2, 4, and 8 kHz for air conduction and 0.5, 1, 2, 3, and 4 kHz, for bone conduction), thus measuring the air-bone gap and audiometric hearing threshold (measured by averaging the hearing thresholds at 0.5, 1, 2, and 4 kHz).

**Figure 1:**
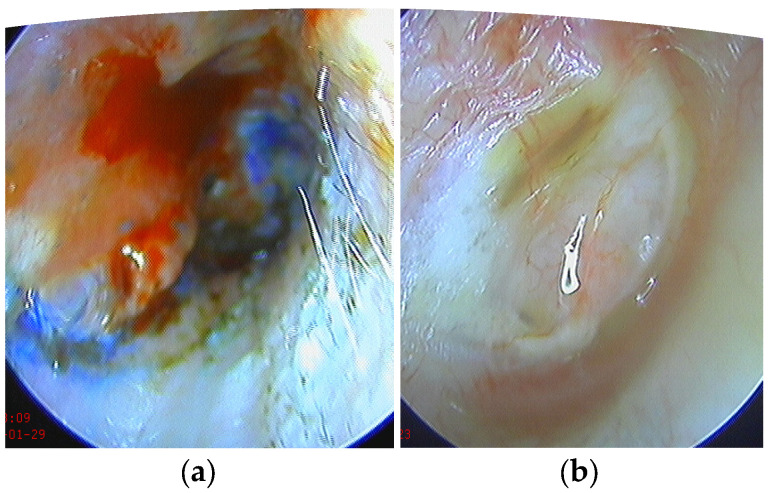
Closure of the perforation with cartilage graft and PRF membrane after removal of the packing of the external ear (**a**) and 1 month postoperative (**b**).

During the tympanoplasty procedure, the surgeons utilized Schambaugh’s incision in the operated ear, creating a tympanomeatal flap. The flap was then elevated to gain access to the tympanic cavity. Following this, any inflamed tissue within the tympanic cavity was removed, and ossiculoplasty was performed if necessary, using either a PORP (Partial Ossicular Replacement Prosthesis) or TORP (Total Ossicular Replacement Prosthesis) prosthesis. The perforation was repaired using a thinned tragal cartilage graft in group 1, while in group 2, both thinned tragal cartilage and a PRF membrane were used. The PRF membrane was larger than the perforation and the cartilage graft, and it was placed using the onlay method, positioned over the sealed perforation and the tympanomeatal flap. The final steps of the surgery involved suturing the Schambaugh’s incision and packing the external ear canal with gauze containing antibiotic ointment. The gauze was kept in place locally for 7 days and then removed under microscopic examination (Figure 1).

Following the completion of the procedure, patients in both groups received broad-spectrum antibiotic treatment for one week. They were also advised to refrain from blowing their nose, getting the operated ear wet, and to attend regular postoperative follow-up appointments.

**Figure 2:**
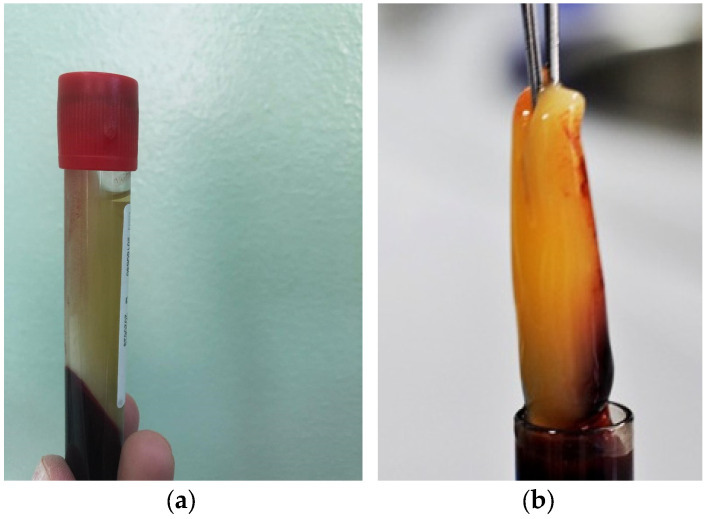
PRF—(**a**) 3 layers of centrifugated peripherical venous blood without anticoagulant and (**b**) fibrin clot.

**Figure 3:**
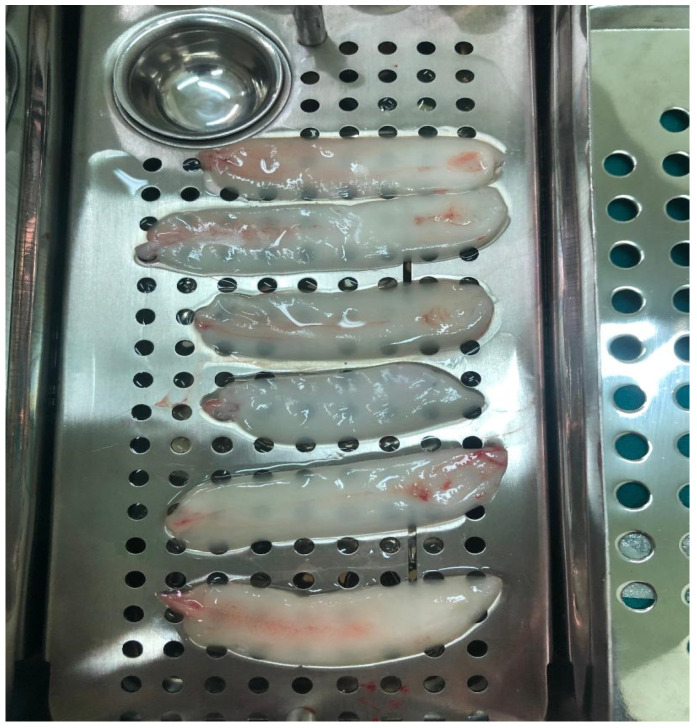
The PRF thin membrane ready to use for ear surgery.

The preparation of autologous PRF was performed prior to initiating the ear surgery. The anaesthesia team collected a 10 mL sample of peripheral venous blood into special tubes without anticoagulant. The collected blood sample was immediately centrifuged for 12 min at 3000× *g* rpm using a Choukroun centrifuge. The centrifugation process resulted in the formation of three distinct layers: a bottom layer containing red blood cells, a middle layer consisting of the fibrin clot, and a top layer consisting of acellular plasma (Figure 2). The PRF clot was carefully extracted and then pressed for 8 min, transforming it into a thin membrane (Figure 3). This PRF membrane was subsequently placed as a plug over the sealed perforation during the surgical procedure.

### 2.2. Statistical Analysis

The statistical analysis was performed using MedCalc^®^ Statistical Software version 20.2.18 (MedCalc Software Ltd., Ostend, Belgium). The data were assessed for normality using the Shapiro–Wilk test. Means and standard deviations or median and 25–75 percentiles were used to describe continuous variables, while frequencies and percentages were used to describe qualitative data. To assess the association between categorical variables, the chi-square test was used. Student *t* test was used to assess differences between groups regarding quantitative variables. The comparisons between preoperative and 12 month’s postoperative measurements were performed using the *t* test for pairs. A *p*-value of <0.05 was considered statistically significant.

Data obtained from medical charts included age, sex, type and localization of perforation (assessed by the endoscopic and microscopic evaluation), type of tympanoplasty performed (tympanoplasty type I-IV), material used (cartilage without PRF and cartilage with PRF), graft status (at 1-3-6-12 months after surgery), and hearing improvement (based on pure-tone audiogram obtained preoperatively and postoperatively at 12 months).

## 3. Results

Our study included a total of 47 patients with chronic tympanic membrane perforations that met the inclusion criteria. All patients were diagnosed with chronic eardrum perforation in both groups, except 1 patient in the PRF group, which presented cholesteatoma, discovered intraoperatively. All the patients had the normal endoscopic aspect of the contralateral ear. All the patients underwent surgical repair of tympanic membrane by closure of perforation using tragal cartilage graft (control group 1) and tragal cartilage graft with platelet rich fibrin membrane (group 2).

Patients in the control were operated on with general anaesthesia and the surgeons performed microscopic tympanoplasty type I-IV with endaural approach, with hearing restauration by PORP/TORP, according to the extent of the lesions of the eardrum and ossicles, using only tragal cartilage graft.

Patients included in the PRF group were positioned the same as the first. The anaesthetic approach was different: 5 patients with local anaesthesia, cases where surgeon decided to use endoscopic tympanoplasty type I, and general anaesthesia for the rest of 15 patients, where they performed microscopic tympanoplasty type II-IV with hearing restauration with PORP/TORP (depending on the ossicles status). Tragal cartilage graft was used along with autologous PRF for all patients. One patient out of the last 15 patients was diagnosed intraoperatively with cholesteatoma requiring tympanoplasty type IV with TORP and graft included cartilage and PRF.

The patients included were 25 males and 22 females wherein the mean age was 42.4 ± 13.8 in the control group and 44.05 ± 9.7 in PRF group, respectively, with a statistically non-significant difference (*p* = 0.6) (Table 1). The most common type of perforation was quasi-total perforation observed, in 40.7% of patients in group 1 and in 60.0% of patients in group 2, respectively, followed by posterior quadrant involved in 33.3% of patients in group 1 and 25.0% in group 2. Large perforations were found in 23 of the 47 patients: in 11 in the control group and 12 in the PRF group, with a statistically non-significant difference (*p* = 0.1, Table 1). No statistically significant differences were observed between groups in terms of gender, age, perforation size type, or tympanoplasty type (all *p* > 0.05). Characteristics of the type of perforation and of tympanoplasty performed are shown in Table 1.

In the present study, postoperative follow-up at 1, 3, 6, and 12 months showed a higher rate of graft survival in the PRF group than the control group as shown in Table 2, but without statistically significant difference in the rates of healing of the tympanic membrane at 1, 3, and 6 months. A successful outcome was observed at 12 months after the surgery in 95.0% of patients from the PRF group, whereas in the control group the success rate was observed in 70.4% of cases. From the PRF group in one patient (5.0%) the closure was not obtained, that was the patient in which we discovered intraoperative the cholesteatoma, but in group 1 the failure of graft, was observed in 29.6% of cases. The difference between the groups at 12 months after the surgery was statistically significant (*p* < 0.05).

Comparisons of improvement in PTA and air-bone gap values of both groups are shown in Table 3. The PTA values improved significantly better in the PRF group one year after the surgery compared to the control group (*p* < 0.001). However, the postoperative air-bone gap values did not differ significantly between groups, although more improvement was observed in the PRF group (*p* < 0.7).

In all cases from both groups there was no complication, additionally, the PRF used in the PRF group was well tolerated and the incisions healed perfectly.

## 4. Discussion

In the study, a PRF membrane was used in combination with autografts to close the perforations in cases of chronic dry eardrum perforation. The PRF membrane contains platelets, white blood cells, and various growth factors, which play an important role in cell proliferation and facilitating the healing process [8]. Since the PRF is autologous, the chances of immunogenic reaction and graft failure are minimal. The process of preparing PRF **is** simple, quick, and cheap, does not increase the time of surgery and is also effective in wound healing and reduces the incidence of infections [3,13,16].

PRF was used in the treatment of tympanic membrane perforations, either alone or with temporal fascia, paper, and gelfoam, and so on [17,18]. Due to its resorbable nature, PRF is commonly used in combination with autografts [12].

In the present study, we had observed higher rates of graft survival in the cartilage graft plus the PRF group than in the cartilage graft without the PRF group. The success of the procedure was indicated by the graft survival and hearing improvement. Closure rates at 1 month were used to evaluate the speed of healing, and closure rates at 12 months were used to evaluate general recovery of the tympanic membrane and hearing improvement. The follow-up at 1, 3, 6, and 12 months showed a higher rate of graft survival in group 2 (cartilage graft + PRF) than group 1.

Our results demonstrate that the utilization of a cartilage graft with PRF leads to a higher success rate, with a graft uptake of 95%, compared to group 1 in closing chronic perforations. These findings are consistent with other studies in the literature. For example, Yadav et al. reported a 95% uptake success rate and an increase of 18.62 dB in hearing improvement in the PRF group, compared to an 85% uptake and 13.5 dB improvement in the group without PRF [19]. Similarly, studies conducted in India have reported a 100% PRF graft uptake with a hearing improvement of 13.75 dB [20]. Gur et al. reported a closure success rate of 93% in the group where PRF and paper were used, compared to 83% in the group using the paper patch technique [21].

Our study provided evidence that there was a significant improvement in hearing outcomes related to postoperative pure tone average in the group with PRF, compared to the group without PRF. The difference in hearing gain between groups was statistically significant. However, there was an improvement in postoperative air-bone gaps value in both groups, the best results were reported in the group with PRF, the difference in air-bone gap value did not differ significantly between groups. The study of Shindy included 50 patients and compared air-bone gap pre- and post-treatment and revealed that the mean air-bone gap significantly decreased after treatment by PRF use in all perforations underlining the low cost, and lack of complications when using PRF [22].

The results of our study showed similar success rates when compared to other studies. Several studies from the literature also reported that in patients with eardrum perforation, successful healing in myringoplasty was seen when involving the use of PRP than without PRF [13,23].

Similarly, the study performed by Gökçe Kütük et al., which included 91 patients with chronic otitis media, demonstrated the superiority of using PRF combined with temporal fascia graft in type 1 tympanoplasty compared with temporal fascia graft alone. The success rate of tympanic membrane repair was 94.4 vs. 74.5%, and also, hearing gain tended to be greater in the temporal fascia graft plus platelet-rich fibrin therapy group, but the difference between groups was not statistically significant [13]. In our study, although in a smaller sample size, where we had performed type I-IV tympanoplasty with endaural approach, and hearing restoration by PORP/TORP, according to the extent of the lesions of the eardrum and ossicles, using tragal cartilage graft plus PRF, wherein we obtained a similar successful healing.

In another study, Riaz N. et al. reported that the graft uptake was greater in patients with PRF (76%) compared to the non-PRF group, but with lesser success rates when compared to other studies and ours (the graft uptake was 95% in the PRF group) and hearing improvement in the PRF group compared to the same studies, but similar to our study [16].

Regarding the use of PRF in ear surgeries, the majority of the studies describe the success rates of the use of PRF in type 1 tympanoplasty, neither in other types of middle ear surgeries nor in different types of tympanoplasty, except the study performed by Garin et al. [3]. They reported a success rate in tympanic membrane repair of 94% (type I, II, III tympanoplasty) using PRF and without any postoperative complications or adverse effect in middle ear packing and also without recurrence or residual disease during second-look revision surgery for cholesteatoma [3]. In addition, the patient from our study discovered intraoperatively with cholesteatoma had no recurrence or residual cholesteatoma or other complication in the healing process after middle ear surgery.

The limitations of our study include a small number of included patients, the lack of previous imaging tests (which could have provided a more comprehensive preoperative diagnostic assessment for cholesteatoma), the inclusion of different types of tympanoplasty procedures, involving a mixture of different surgical techniques (both classic approach and endoscopic), and the absence of an evaluation of the early healing time in the PRF group compared to the non-PRF group.

## 5. Conclusions

The management of chronic perforations with PRF has shown promising results and an increased rate of autograft survival in tympanoplasty surgery with hearing recovery. PRF can be used as an effective material for patients with chronic perforations of the tympanic membrane.

## Figures and Tables

**Table 1:** Characteristics of the eardrum perforations in both groups.

Variable		Control Group	PRF Group	*p*-Value
Age		42.4 ± 13.8	44.05 ± 9.7	0.6
Sex	M	12 (44.4%)	13 (65.0%)	0.2
	F	15 (55.6%)	7 (35.0%)	
Perforation * Type	1	5 (18.5%)	-	0.1
	2	9 (33.3%)	5 (25.0%)	
	3	2 (7.4%)	3 (15.0%)	
	4	11 (40.7%)	12 (60.0%)	
Tympanoplasty	I	12 (44.4%)	5 (25.0%)	0.5
	II	5 (18.5%)	5 (25.0%)	
	III	4 (14.8%)	4 (20.0%)	
	IV	6 (22.2%)	6 (30.0%)	

* Type 1 = anterior; 2 = posterior; 3 = central; 4 = large—almost complete.

**Table 2:** Graft survival in both groups.

Variable	Closure Perforation	Control Group	PRF Group	*p*-Value
1 Month	Yes	24 (88.9%)	20 (100%)	0.2
	No	3 (11.1%)	0 (0.0%)	
3 Month	Yes	21 (77.8%)	19 (95.0%)	0.2
	No	6 (22.2%)	1 (5.0%)	
6 Month	Yes	20 (74.1%)	19 (95.0%)	0.1
	No	7 (25.9%)	1 (5.0%)	
12 Month	Yes	19 (70.4%)	19 (95.0%)	0.05
	No	8 (29.6%)	1 (5.0%)	

**Table 3:** Functional results in both groups.

Variable			Control Group	PRF Group	*p*
PTA-value	Preoperative		31.2 (27.5; 40)	32.5 (28.7; 38.6)	0.001
	12 months postoperative		22.5 (17.5; 31.2)	15 (11.8; 21.8)	
Air-bone gap	Preoperative		20 (15; 22.5)	13.7 (11.2; 18.4)	0.7
	12 months postoperative		5 (2; 10)	2.5 (1.2; 3.5)	
	Preoperative	500	21(16; 23.5)	16.2 (12.5; 19.6)	0.8
	12 months postoperative	500	6 (2.7; 11.2)	3 (1.7; 4)	
	Preoperative	1000	20.5 (15.5; 22.8)	13.7 (11.2; 19)	0.7
	12 months postoperative	1000	5.5 (2.5; 10.5)	2.7 (1.5; 3.7)	
	Preoperative	2000	19.8 (14.8; 21)	11.8 (10.3; 17.8)	0.5
	12 months postoperative	2000	4.5 (2.2; 10)	2.5 (1.2; 3.5)	
	Preoperative	4000	17.5 (13; 19.5)	10 (10; 15.6)	0.4
	12 months postoperative	4000	3.5 (1.5; 9)	2 (0.7; 3)	

Data represent median and 25–75 percentiles values (in decibels).

## Data Availability

The data used to support the findings of this study are included within the article.
